# Evangely, the subcategorization has been announced in the 2023 Bethesda system for reporting thyroid cytopathology: let bygones be bygones in thyroidology!

**DOI:** 10.1590/1806-9282.20231511

**Published:** 2024-04-22

**Authors:** Ilker Sengul, Demet Sengul

**Affiliations:** 1 Giresun University, Faculty of Medicine, Division of Endocrine Surgery – Giresun, Turkey.; 2 Giresun University, Faculty of Medicine, Department of General Surgery – Giresun, Turkey.; 3 Giresun University, Faculty of Medicine, Department of Pathology – Giresun, Turkey.

Dear Editor,

The 2010 TBSRTC, first edition, was initially proposed in Bethesda, Maryland, USA, in 2007, providing thyroidologists a standardized, reporting system for thyroid FNA, in Volume 19, Thyroid^1^. Wielding TBSRTC has also been endorsed by the 2015 American Thyroid Association management guidelines^2^ through the delicate papillon gland^3-7^. A special 2½-h symposium was moderated by Ali and Vielh at ICC in Pacifico Yokohama, Japan, on 28 May–01 June 2016^8-10^. The 2017 TBSRTC, second edition, was then published in Volume 27, Thyroid, by amendment of indeterminate cytology^11^. However, (re)appraisal for atypia of undetermined significance (AUS) or follicular lesion of undetermined significance (FLUS), category III, is still one of the most challenging issues in thyroidology as well as for Endocrine and Head & Neck Radiologists^12-17^. To this end, we emphasized whether it is essential to maintain category III as a unique one in February 2021, Volume 67, Rev Assoc Med Bras^18^. In October 2021, we declared blurred lines for managing thyroid nodules in the era of category III in a possible forthcoming TBSRTC, third edition. Of note, we postulated the so-called subdivision in category III as (i) IIIA: AUS/FLUS without nuclear atypia (AUS/FLUS w/o NA) and (ii) IIIB: AUS/FLUS with nuclear atypia (AUS/FLUS w/ NA) in Volume 67, Rev Assoc Med Bras^19^. Finally, we have currently recommended working with subsets to resolve the ongoing debate on "indeterminate cytology," similar to "intermediate suspicion" in Radiology, in Ultrasonography with a submission date of June 08, 2023^20^.

Evangely, just 1 month later, the third edition of this lexicon, the 2023 TBSRTC, has been announced after two former successful editions by Ali et al., on July 08, 2023, in Thyroid. They have stated that the 2023 TBSRTC discontinues the term "FLUS" to avoid confusion with reporting terminology; henceforth, only "AUS" will be used. Of note, the up-to-date third edition declared the subcategorization of category III as (i) AUS-NA and (ii) AUS-other. Today, this two-tiered subclassification^21^ has confirmed our previous recommendation for subdivision: (i) AUS w/ NA and (ii) AUS w/o NA in Volume 67, Rev Assoc Med Bras^19^.

Hic et ubique terrarum, NAs have non-negligible clues in these nodules with indeterminate cytology. E fructu arbor cognoscitur. Eventually, the subcategorization has been announced after a long expectancy. Evangely, let bygones be bygones!^21^ We are deeply grateful to Cibas and Ali, founders of this crucial thyroid lexicon stating "just keep study" instead of "just keep stu(ea)dy", particularly for the indeterminate era in thyroidology. Novi sub sole, subdivision is no more debatable, in thyroidology, as we kindly have advocated in Rev Assoc Med Bras^22^.
